# Obesogenicity of food in the informal food retail environment of low- and middle-income countries: a systematic review

**DOI:** 10.1136/bmjgh-2024-017783

**Published:** 2025-12-19

**Authors:** Trish Muzenda, Jean Adams, Lambed Tatah, Muhammad Rabiu Balarabe, Tolu Oni

**Affiliations:** 1 MRC Epidemiology Unit, University of Cambridge, Cambridge, UK; 2 School of Public Health and Family Medicine, University of Cape Town, Rondebosch, South Africa

**Keywords:** Systematic review, Global Health, Nutrition, Public Health

## Abstract

**Background:**

Evidence on food environment obesogenicity has largely focused on the formal sector in high-income countries, overlooking informal food retail environments in low- and middle-income countries (LMICs).

**Aim:**

To synthesise current evidence on the obesogenicity of foods in informal food retail environments in LMICs.

**Methods:**

A systematic literature search across four academic databases: Scopus, Web of Science, EBSCOhost (Global Health) and EMBASE, using predefined inclusion and exclusion criteria.

**Results:**

Thirty studies met the inclusion criteria and were included in the synthesis. Findings indicate the pervasive availability of unhealthy or more obesogenic alternatives at informal food outlets located at transport stops, public markets, schools and neighbourhoods. The results also highlight the hybrid nature of informal food environments in LMICs, where both healthier and obesogenic options. For example, at transport stops, both fresh produce and high-calorie snacks are readily available. These findings illustrate the complex interplay between food availability, customer preferences and dietary outcomes.

**Conclusion:**

This review highlights gaps in understanding the informal food environment and the need for further research to address its complexity. The hybrid nature of this environment calls for strategies that incentivise food outlets to improve the healthfulness of their offerings. Policymakers and public health practitioners should consider tailored interventions to support healthier food choices within informal food retail settings.

**WHAT IS ALREADY KNOWN ON THIS TOPIC**

- Research on obesogenic food environments has focused on formal settings in high-income countries, with little attention to informal food environments in low- and middle-income countries (LMICs).

**WHAT THIS STUDY ADDS**

- This study highlights the hybrid availability of both obesogenic and healthier food options in informal food environments in LMICs.

**HOW THIS STUDY MIGHT AFFECT RESEARCH, PRACTICE OR POLICY**

- The study underscores the need for targeted research and policy interventions to incentivise informal food retailers to increase the availability of healthier food options and reduce the prominence of obesogenic choices.

## Introduction

 Overweight and obesity are pressing public health challenges in low- and middle-income countries (LMICs).^1^ The rising incidence of these conditions is driven by upstream factors including urbanisation, the nutrition transition, globalisation and liberalisation of food systems and trade.^2^,^5^ These factors converge to create obesogenic food environments, dominated by energy dense processed and ultra-processed foods (UPF),^1 2^ which promote obesity at both individual and population levels.^6 7^ Evidence suggests that the widespread availability of UPFs, typically high in fats, salt and sugars, contributes significantly to the increasing prevalence of obesity.^2 3 5^ Such foods are designed to be highly palatable and to override the body’s natural satiety signals, promoting overconsumption.^8 9^ Additionally, changes in the preparation of traditional foods, particularly the increased use of cheap vegetable oils, have substantially elevated fat intake and total energy consumption.^10^

The influence of food product availability on dietary patterns and related health outcomes, including overweight and obesity, can be examined through the concept of food retail environments. These are defined as "the interface where people interact with the broader food system to acquire and consume foods".^7 11^ Food retail environments comprise five dimensions: availability, accessibility, affordability, acceptability and accommodation.^12 13^ Availability refers to the supply of options, such as the presence of specific outlets or products. Accessibility is geographic, capturing the location of food sources and the ease with which they can be reached. Affordability considers food prices in relation to economic factors, such as household income or price indices. Acceptability reflects people’s perceptions and attitudes towards the local food environment. Lastly, accommodation examines how well food outlets align with customer needs, including factors like store hours and accepted payment methods.^12^

In LMICs, the food retail environment consists of two sectors, formal and informal.^14^,^17^ The formal retail sector primarily includes registered businesses offering a diverse range of food products, from raw ingredients to prepared foods. Examples include supermarkets^18^ and grocery stores.^19^ The product offerings within the formal sector have been well documented, with globalisation and manufacturing influencing the types of foods available in LMICs.^18 20^ In contrast, the informal food retail sector primarily consists of small-scale outlets operating from locations such as street pavements, mobile food carts, temporary physical structures and food markets.^15 21 22^ These outlets are often not formally registered or licensed with government authorities but may hold permits allowing them to operate in specific locations. Due to their small scale, individual outlets typically offer a narrower range of products and sell items in smaller unit sizes.^16^ At the aggregate level, these outlets collectively provide access to a diverse range of food products. The scale of the informal food retail sector in LMIC is reflected in studies examining consumer market share relative to formal outlets. In Kenya, Nicaragua, Zambia, Thailand and Mexico, informal food retail outlets dominate food retail markets, accounting for at least 60% market share.^23 24^ Despite these distinctions, the formal and informal food retail sectors maintain a symbiotic relationship.^14^ The informal sector frequently sources products in bulk from supermarkets and wholesalers in the formal sector, either repackaging them into smaller units or using them as ingredients for food preparation and resale.^14^

The informal food retail sector offers several key advantages. These outlets are conveniently located near customers, reducing the need for transportation and improving physical access to food.^25^ Additionally, food prices in the informal retail sector are comparatively lower compared with the formal sector.^21 26^ In some instances, outlets offer small credit facilities to regular customers,^16 19^ enhancing short-term affordability. The informal retail sector also plays a vital role in supplying healthy whole grains, legumes, fruits and vegetables, thereby contributing significantly to household food diversity.^16 21 26^ Furthermore, informal food outlets provide food items that align with the sociocultural preferences of their customers, adding to their acceptability.^16 26^

These advantages collectively emphasise the significant role played by the informal food sector in enhancing food access, affordability, acceptability and security for LMIC communities. However, existing literature^27^ lacks a comprehensive analysis of obesogenic foods in informal food retail environments, including an exploration of available food types and the factors influencing food purchasing choices. This systematic review aims to explore this gap by examining current evidence concerning the obesogenicity of foods in the informal food retail environment in LMICs.

## Methods

We conducted a systematic review^28^,^30^ to synthesise evidence on the obesogenicity of foods in the informal food retail environment in LMICs according to the Preferred Reporting Items for Systematic Reviews and Meta-Analyses criteria.^31^

### Record search and identification

The first step involved identifying search terms to identify studies examining the obesogenicity of foods within informal food retail environments. The search strategy consisted of the following terms—‘obesity’, ‘informal’, ‘food environment’ and ‘low- and middle-income countries’. The detailed search strategy is available in online supplemental file 1.

We designed a broad and sensitive search strategy to capture relevant studies that may not explicitly use technical descriptors such as ‘ultra-processed’, ‘high-fat’ or ‘energy-dense’, but that nonetheless engaged with obesogenic food environments. To maintain rigour, we subsequently applied more targeted inclusion criteria during screening (described below) to focus on studies examining the nature, availability or implications of obesogenic foods within informal retail settings in LMICs. Search terms were systematically applied across four databases: Scopus, Web of Science, EBSCOhost (Global Health) and EMBASE in June 2023 and again in April 2024 to capture recent publications.

### Screening

Research articles were included based on the following inclusion criteria: the study reported on empirical research findings and explored aspects of obesogenic foods—typically high in fats, salts and energy density—within informal food retail environments. Studies could use any tools or techniques, whether quantitative or qualitative, to evaluate the informal food retail environment.

There were no restrictions on language or publication date, provided the research data were collected in an LMIC setting. We excluded studies conducted in high-income country contexts; those that did not examine any aspect of the informal food environment or its relationship to diet, nutrition status, overweight or obesity and papers that were not empirical in nature, such as editorials, commentaries or reviews.

Two reviewers (LT and TM) independently screened all records using the Covidence web-based screening platform.^32^ Conflicts were addressed through discussions between the reviewers to reach a consensus. 53 articles advanced to full-text review and were screened by LT, MRB and TM. Each article was independently assessed by two reviewers, and any conflicts were resolved through discussion to achieve consensus. The third reviewer, not having previously reviewed the article, provided input for consensus. 30 articles met the inclusion criteria and underwent data extraction and quality appraisal.

### Data extraction and analysis

The data extraction template captured comprehensive information, encompassing general article details (such as title, country and setting), study objectives, design and sampling strategies; data collection methods; key aspects of the informal food retail environment and inferences about the obesogenicity of the environment. All studies were allocated to the food environment domains—availability, accessibility, affordability, acceptability and accommodation—based on our interpretation of the study content, in line with the descriptions by Caspi *et al*^12^ and Penchansky and Thomas.^13^ Data were extracted by two reviewers and checked for consensus. Information gathered from data extraction was further analysed and narratively synthesised in line with the aims.

We assessed the quality of research evidence using the validated Appraisal tool for Cross-Sectional Studies (AXIS)^33^ for studies that used a quantitative cross-sectional design. Qualitative studies were not formally assessed using AXIS. Instead, we considered their methodological transparency, including clarity of aims, sampling and data collection methods, during the narrative synthesis.

## Results

**Figure 1:**
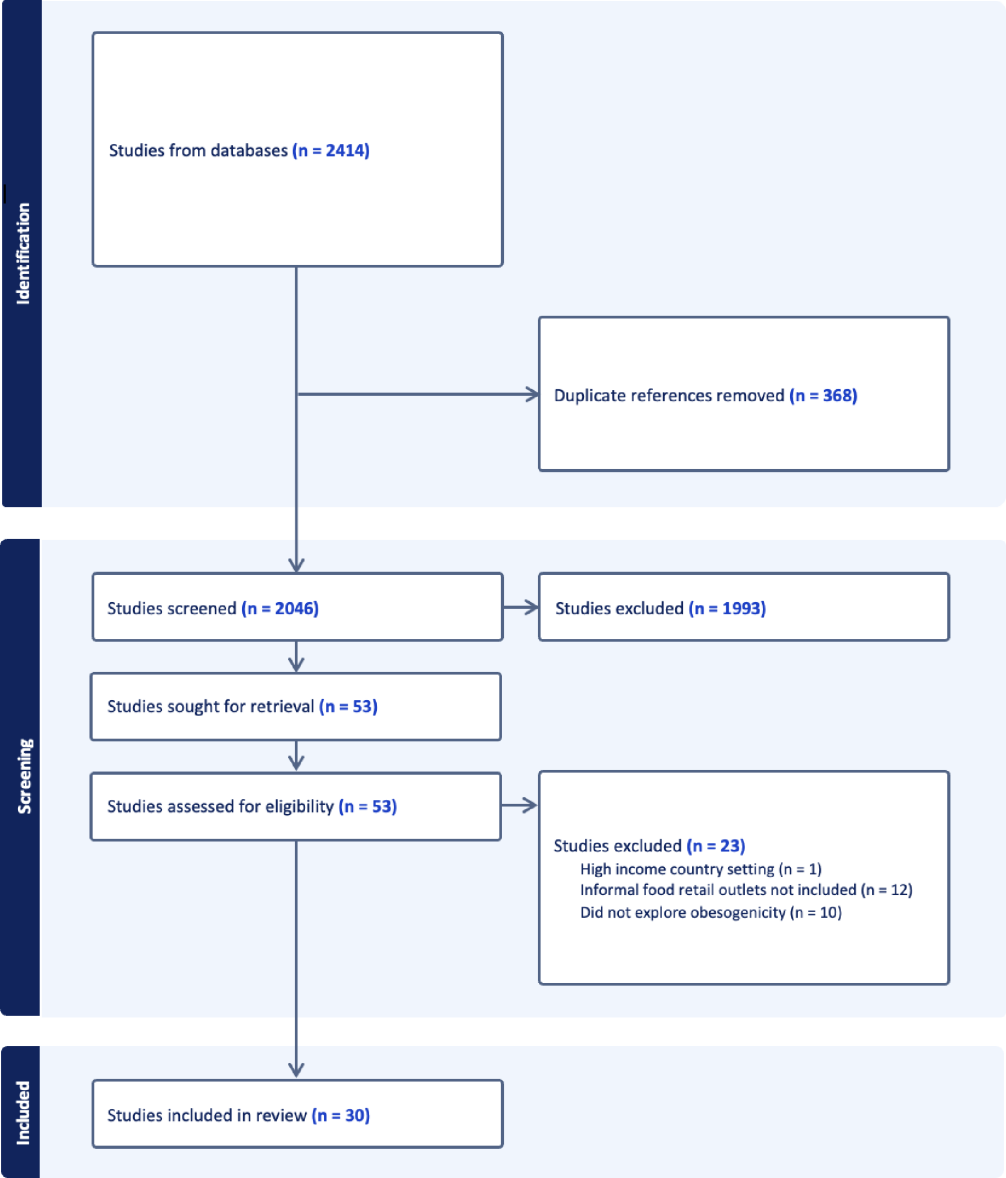
Preferred Reporting Items for Systematic Reviews and Meta-Analyses criteria flow diagram.

The initial systematic search in June 2023 yielded 2050 records, of which 235 were duplicates. An updated search conducted in April 2024 retrieved an additional 364 records, with 133 duplicates. After removing duplicates, a total of 2046 records were screened by title and abstract. Of these, 53 articles were reviewed at full text, and 30 studies met the inclusion criteria and were included in the review (figure 1).

30 studies met the inclusion criteria (figure 1). These studies were distributed across five LMIC regions: Africa (n=18),^34^,^51^ South Asia (n=4),^52^,^55^ Latin America (n=3),^56^,^58^ Central Asia (n=3)^59^,^61^ and Eastern Europe (n=2).^62 63^ Within the African region, evidence was from seven countries, with almost a third of these studies being conducted in South Africa (n=5).^37 38 43 45 47^ Of the included studies, 27 used a quantitative cross-sectional design. The remaining three used qualitative designs, with data collected at a single point in time. A summary of included studies is provided in online supplemental file 2.

In included studies, the obesogenicity of the informal food environment was assessed across three of the five dimensions: availability (n=26), accessibility (n=26) and acceptability (n=4). The dimensions of accommodation and affordability were not explicitly explored in any study.

Overall, the quality of the included studies was good. Most were assessed as high quality based on core AXIS criteria (online supplemental file 2). Three studies were rated as moderate quality due to specific methodological concerns. Two studies^61 63^ relied on non-validated observation methods to estimate the body mass index (BMI), which may introduce measurement bias. One study^43^ reported potential response rate bias related to social tensions affecting informal food vendor participation.

### Availability

All studies (n=26) assessing the availability of obesogenic food in informal food outlets reported a pervasive availability across diverse settings.^34^,^4042^ The proportion of obesogenic foods, in relation to total food items sold, varied based on factors such as geographical location and local sociocultural context.

Evidence from studies that conducted nutritional assessments of food items sold in informal food outlets highlights a high availability of energy-dense offerings.^37 38 54 59 61 62^ In Moldova,^62^ 60.3% (n=328) of street vendors were reported to sell processed or UPFs, with a mean energy density of 430 kcal/100 g serving. This is significant considering that 100 g of this food constitutes about a fifth of the recommended daily caloric intake for women (2000 kcal) and just under a fifth for men (2500 kcal).^64^ In South Africa, Faber *et al* noted that popular corn-based snacks sold in and around 94% (n=36) of schools had energy densities exceeding 478 kcal/100 g.^37^ Another study found that while fast food was available in all retailers (n=18) surveyed, 12 out of 15 assessed foods had energy densities ranging from 280 to 364 kcal/100 g.^38^ Additionally, a multicity study^61^ conducted in Tajikistan, Kyrgyzstan, Turkmenistan and Kazakhstan revealed that approximately one-third (31.9%, n=714) of customers purchased UPF or beverages, with individual items having a high energy density of 581 kcal/100 g. Additionally, the study also found that food sold from informal ‘traditional’ food outlets had a high fat content such that a single street food purchase could provide a third of the recommended daily limit of saturated fat intake.^61^

Research investigating the relationship between frequent street food consumption and BMI revealed a positive correlation between these factors. In Tanzanian^40^ and South African^47^ studies, a reported positive association emerged between street food consumption and the BMI of regular customers at both public markets and transport stops. Likewise, a Mexican study^58^ examining the link between student BMI and the types of foods sold by mobile food vendors found that 85% of outlets primarily offered unhealthy food products such as tamales, ice cream and pizzas. Additionally, the study observed a positive correlation between the number of informal mobile food vendors near schools and student BMI.

While nearly all studies reported on the availability of obesogenic food, they also unveiled a hybrid nature of the informal food environment. This duality is characterised by the coexistence of more obesogenic foods alongside healthier options. This suggests a hybrid food environment where healthier choices and obesogenic foods coexist along a continuum. For example, in Mozambique, despite 71% of food vendors offering healthy options, 59% also provided UPFs.^48^ Use of the obesogenic risk indicator in South Africa, Kenya and Ghana particularly highlighted the dual nature of obesity risk within the informal food environment, as both obesity-promoting and obesity-protective foods were found to coexist in the same retail outlets. However, the prevalence of each category depended on contextual factors, particularly the types of foods typically sold by informal retailers. For instance, when comparing data from Ghana and South Africa,^43^ researchers observed that Ghana had a predominance of low obesogenic risk foods, possibly due to a higher concentration of fresh produce markets. In contrast, South Africa exhibited a higher prevalence of outlets selling high obesogenic risk foods.

A hybrid informal food environment was also observed around schools. In South Africa, Faber *et al*^37^ noted the availability of both healthy and obesogenic foods sold by informal retailers operating within and around schools. Similar findings were noted in Kenya, where school food environments offered a mixture of both healthier and less healthy food alternatives to varying degrees.^36^ Notably, food outlets situated around schools in low-income areas exhibited a greater availability of obesogenic foods compared with healthier alternatives.^36^ In Ghana, the modified Retail Food Environment score indicated that a larger proportion of food outlets (53.4%) in and around schools offered healthier items compared with less healthy options.^46^ However, despite this, 40.6% (n=1124) of observed food purchases made by pupils during school breaks consisted of high-energy snacks and beverages.^46^ In their qualitative inquiry, Pehlke *et al*^57^ observed that the offerings in informal food kiosks in and around schools predominantly comprised sandwiches, calorie-rich snacks and sugar-sweetened beverages, aligning with perceived student preferences. While fruits were also available, they were not as popular among the students.

### Accessibility

None of the included studies explicitly measured the accessibility of obesogenic foods by assessing the location of informal food retail outlets relative to the location of clients. Inferences about the physical access to obesogenic foods were place-based and drawn from the choice of sampling sites used in these studies—public markets (n=7),^4041 59^,^63^ transport stops (n=2),^47 48^ schools (n=8),^34 36 37 45 46 55 57 58^ university (n=1)^44^ and neighbourhoods (n=8).^3538 39 42 43 52^,^54^ For example, studies conducted in Kenya,^35^ Pakistan^53^ and India^54^ examined the spatial distribution of food outlets as a proxy measure for the ease of access residents had to various types of food within those neighbourhoods. As noted under the availability domain, there was varying accessibility of both less and more obesogenic foods across the informal food retail outlets sampled in all the studies.

One qualitative study from South Africa using the photovoice approach^45^ aimed to explore access to obesogenic foods within primary school students’ home, community and school food environments. An interesting finding from this study was the prevalence of ‘informal home shop vendors’, operating from within their homes without a physical structure comparable to other informal food outlets. These outlets were often located close to students’ homes and sold snacks and fizzy drinks deemed to be more obesogenic in nature. Students reported purchasing food from these outlets rather than from other informal food outlets near their homes.

### Acceptability

Four studies investigated food acceptability, using qualitative (n=3) and quantitative (n=1) inquiry to explore perceptions regarding the types of foods sold by informal food retailers^50 55 57^ and barriers in the supply of and demand for healthy food.^41^

Evidence from Indonesia^55^ and Guatemala^57^ was derived from interviews with school educators, students and street food vendors. These studies examined the array of foods available within the school food environment. In both cases, the informal food retail environment was acknowledged as an important source of refreshments for students. However, there was a consensus that the food items sold around schools were calorie-dense and generally unhealthy. Yet, school authorities in the Indonesian context did not perceive these as issues within their jurisdiction to address. Their influence was limited to changes in the food sold inside the school premises.

Another study explored the challenges associated with implementing a healthy plate model in Dar es Salaam, Tanzania.^41^ Interviews with informal food retailers selling cooked foods revealed obstacles in providing healthier food options and portion sizes in line with the healthy plate model.^41^ Vendors mentioned that customers preferred smaller portions of vegetables compared with starchy items and meat and expressed concerns about the capital costs and pricing of plates aligned with the healthy plate model. Even when they were aware of and supportive of the healthy plate model, these factors deterred them from implementing it. The small scale of their businesses meant they lacked financial resources to take risks that conflicted with their perceptions of their customers’ food preferences. In another Tanzanian study,^50^ using a semistructured questionnaire, perceptions and attitudes toward healthy and unhealthy meals sold by street food vendors were assessed. Findings revealed that 96% of street food vendors recognised the link between unhealthy diets and diseases, with 99% acknowledging the negative impact of excessive fats and oils on health. While 99% agreed that street food vendors should offer healthy meals to consumers and 89% claimed to do so, only 74% emphasised the importance of considering meal energy content.

## Discussion

We conducted the first systematic review synthesising current evidence on the obesogenicity of foods within informal food retail environments in LMIC contexts. This review uniquely addresses dimensions of availability, accessibility and acceptability in settings that are often overlooked in the literature. Our findings suggest that obesogenic food options are available alongside healthier alternatives, indicating a continuum rather than a stark dichotomy of obesogenicity of available food. Place-based sampling techniques provided insights into the objective accessibility of food within informal sectors, specifically in public spaces such as transport stops, public markets, schools and neighbourhoods. Furthermore, qualitative methods highlighted the activity of informal food vendors operating from home without physical structures, underscoring the limitations of conventional measures of food accessibility. Studies assessing the perceived acceptability of food to customers showed that informal food retailers were aware of the calorie density and overall healthfulness of their offerings but continued to serve these foods due to perceived customer demand.

Studies focusing on availability^34^,^4042^ emphasised the pervasive availability of obesogenic food items within informal food retail settings. This trend was evident in the availability of energy-dense foods, elevated levels of fat and oil in food and foods and beverages known to be obesogenic. The availability of these items, both in informal and broader food environments, aligns with the expected outcomes of the nutrition transition in developing countries.^3^ This transition involves a shift from ‘traditional diets’ to ‘western diets’, characterised by heightened consumption of refined carbohydrates, added sugars, fats and animal-source foods.^2 3^

Consequently, the increased availability of obesogenic food creates a positive feedback loop with customer demand. As dietary patterns shift towards these foods, their availability in the informal sector is likely to increase. This scenario is particularly evident in the informal food retail sector, where, apart from small kiosks, outlets typically offer a limited range of food products and small profit margins, leaving little room for risk taking.^15 16 21^ Additionally, while all food retailers are highly attuned to customer demand to enhance sales and maximise profits, the informal sector may be even more responsive due to direct interactions between business owners and customers. This direct interaction allows business owners to receive immediate feedback related to their food products through direct inquiry. Although none of the studies explicitly addressed the accommodation domain, these insights suggest relevant information about how businesses respond to and accommodate customer demand.

This reality highlights the complex relationship between food availability, consumer demand and dietary patterns. It also encourages consideration of whether increased availability of healthier alternatives would drive their supply, purchase and consumption, similar to the patterns observed with unhealthy, more obesogenic foods.^2^ On the other hand, there is the question of whether customers showing more preference for healthier options would influence informal retailers to stock more of these alternatives. These questions underscore the intricate relationship between food availability, consumer demand and the potential impact on dietary patterns.

Alongside the noticeable availability in obesogenic food items, findings suggest that informal food retail outlets offer a hybrid mix of both healthy and unhealthy foods. The relative market share of each type varies depending on the context.^36 37 43 46 48 57^ Consequentially, customers visiting these outlets are presented with a spectrum of choices, ranging from health-promoting options to obesogenic ones. Choosing which foods to buy is a complex matter, influenced by factors such as sociocultural norms, health awareness, acceptability and affordability. The affordability of food items adds another layer to this complexity. Evidence suggests that globalisation and trade liberalisation in food production and distribution have increased the availability of cheaper obesogenic foods in urban areas of LMICs.^265^,^67^ For instance, Popkin *et al* highlighted how changes in edible oil production have made vegetable oils more affordable, allowing individuals in LMICs to increase their energy intake despite modest incomes.^3^ This has facilitated the widespread use of vegetable oils in informal food settings, where frying becomes a common and affordable method of food preparation. This further contributes to the availability of cheaper energy-dense foods in the informal food environment.

The physical accessibility of obesogenic foods from informal food retailers was inferred by assessing their availability in public spaces regularly visited by the target population, including markets,^4041 59^,^63^ transport stops^47 48^ and schools.^34 36 37 45 46 55 57 58^ This aligns with literature, which shows that informal food retail outlets often cluster around transport stops, major shopping centres, public markets and schools, ensuring a consistent flow of foot traffic for food purchasing.^15 16^ Place-based sampling strategies, tailored to the types of customers frequenting these areas, offer a nuanced understanding of physical access. This is because estimates based solely on the distance between residences and food outlets may not fully capture where people spend their time. Many people buy a portion of their food from outlets in workplaces, transit areas or larger food markets outside their neighbourhood.^68 69^ This underscores the importance of considering individuals’ activity spaces^70^—various locations they visit in their daily lives. People’s daily routines and movements shape exposure to different food environments. Evidence from high-income countries highlights the difference between objective and perceived measures of household food access,^71 72^ with some households choosing to bypass nearby stores or travel outside their neighbourhood for food perceived as more suitable.^71 72^

To further enhance this evidence, studies measuring food accessibility in LMICs could benefit from complementing quantitative data with qualitative inquiries to better understand the nuances of real-life access to obesogenic food items sold by informal food retailers. For example, a South African study^45^ highlighted these nuances by having school-going adolescents photograph their lived food environment. This exercise revealed an additional type of food outlet—‘home shops’—where adolescents purchased food and snacks on their way to and from school.^45^ These outlets, which lack a formal physical structure and are situated inside homes, were not identified in other studies reviewed here. Their significance as key locations for accessing energy-dense snacks and sugar-sweetened beverages might have been overlooked in purely quantitative analyses. These findings underscore the necessity of employing mixed methods that integrate both subjective behaviours and objective place-based measures to fully understand the physical accessibility and range of food offerings at informal food retail outlets.

Three studies explored informal food retailers’ perceptions of obesogenic food availability.^41 52 57^ These studies acknowledged the greater availability of obesogenic food options compared with healthier alternatives and recognised the potential negative impact on health. However, informal traders expressed limitations in their ability to address these concerns and promote healthier food choices, as they are overly sensitive to customer preference. For instance, despite the positive concept of the healthy plate model in Tanzania encouraging great consumption of vegetables and less of starchy foods, retailers noted that customers still preferred larger quantities of starchy and meat items and smaller portions of vegetables.^41^ Consequently, retailers find themselves constrained by the demand for less healthy options to sustain their livelihoods. This highlights the complexities of improving access to healthier food alternatives in the informal food environment. Initiatives aimed at educating and supporting both retailers and consumers could be crucial in fostering positive changes in such contexts. Valuable insights can be drawn from initiatives like the Healthier Catering Commitment in London,^73^ which recognises restaurants making efforts to improve the healthiness of their food offerings. Implemented changes that are considered imperceptible to customers, such as using healthier ingredients like low-fat mayonnaise, offering smaller portions at reduced costs and reducing excess fat in fried foods, have shown varying levels of success.^73^

From a methodological perspective, this review also highlights that the majority of studies examined focused on the availability (n=26) and accessibility (n=26) of foods within informal food environments. This emphasis mirrors findings from previous global and LMIC reviews, which have shown that research on food environments tends to privilege availability and accessibility measures.^27 74 75^ A scoping review across LMICs found that most empirical work centred on availability (n=63), while dimensions such as affordability (n=14) and desirability (n=21) were less frequently addressed.^27^ Comparable patterns are evident in high-income settings. A recent systematic review found that 70% of extracted measures focused on food availability and 23% on accessibility, with only 2% addressing price and 1% affordability.^75^ In the UK, the most common domain studied was availability (n=100, 32%), followed by product characteristics or quality (n=94, 30%). Few studies examined affordability (n=16, 5%) or sustainability (n=19, 6%).^74^ These patterns likely stem from the relative ease of quantifying availability and accessibility through observational or spatial methods, compared with more subjective dimensions such as affordability, accommodation or acceptability. Methodological and resource constraints, including limited data systems and the absence of validated tools for measuring experiential or perception-based factors, have also reinforced this imbalance.^26^ Addressing these gaps will require future studies to adopt broader or mixed methods approaches that capture the full spectrum of influences shaping informal food environments.

## Strengths and limitations

To our knowledge, this study is the first comprehensive attempt to synthesise knowledge about the obesogenicity of foods within the informal food retail environment in LMICs. A notable strength of this review is the lack of restrictions on the study design of the included articles, which allowed for a broad perspective on the current evidence regarding the obesogenic nature of the informal food retail environment in LMICs.

Additionally, in designing the search terms for the review, both academic and grey literature were searched to identify terms commonly used to describe informal food retail outlets. This is particularly important due to the complexity of defining informal food retail outlets across different contexts. The reviewed studies used a wide range of terms to describe these outlets, including ‘street food’,^40 52^ ‘mobile food vendors’,^53 58^ ‘hawker’^35 46^ and ‘street trade’.^43 48^ This diversity underscores the context-specific nature of these outlets and the challenge in providing a universal definition.

There was some conflation in the terminology used to describe the healthiness of the food environment, with terms such as obesogenic, obesity-promoting and less healthy often used interchangeably. We ensured that each study was interpreted according to its specific definitions, which were then used in our analysis. This approach highlights a broader issue in food environment research, where there is a need for standardised classifications of healthy versus unhealthy food, leading to some heterogeneity in the field. Additionally, it is important to note that our review may not have fully captured literature on healthier food options sold in informal sectors, which could provide a different perspective from the one reported in the studies we included.

Despite our comprehensive search strategy, we acknowledge several limitations in this study. The terminology used to delineate the types of informal food retail outlets or obesogenicity in our search may not have been exhaustive. While the studies presented here offered valuable insights into the accessibility of obesogenic foods at informal outlets located in specific areas, the place-based sampling techniques used did not allow for comparisons with less accessible locations, such as home-shops. This limitation should be considered when interpreting the findings. Additionally, the conceptual framework guiding this review does not explicitly include dimensions such as marketing and promotion, which are recognised in other models as additional domains of the food environment.^76^ This reflects a broader issue in food environment research, where multiple conceptual frameworks coexist and there remains limited consensus on which dimensions should be prioritised or how best to operationalise them.^26 27^

All studies included in this review were cross-sectional, limiting our ability to comment on temporal changes in the obesogenicity of the informal food environment. Moreover, there was limited data on the relationship between exposure to food in the informal sector, dietary habits and overweight and obesity outcomes. Future studies should focus on this area to comprehensively demonstrate the significance of informal food environments as predictors of health outcomes.

Lastly, our search terms were set in English, which may have led to the exclusion of relevant articles not indexed in English, despite the databases included in our search encompassing a wide range of languages.

## Conclusions

This review of evidence on the obesogenicity of foods within informal food retail environments in LMIC reveals a multifaceted landscape. The studies highlight the pervasive availability of unhealthy or more obesogenic alternatives at informal food outlets in locations such as transport stops, public markets, schools and neighbourhoods. Additionally, our findings emphasise the hybrid nature of these environments, where both healthier and obesogenic options are offered. Evidence also points to the reluctance of informal retailers to shift away from obesogenic foods, driven by customer preferences. These findings illustrate the intricate relationship between food availability, customer preferences and their impact on dietary outcomes.

This review highlights the need for further research to better understand the complexities of the informal food environment across its five dimensions: availability, accessibility, affordability, acceptability and accommodation. Notably, there is a near absence of evidence addressing accommodation and affordability, indicating a critical gap in the literature. Improved methodologies are essential for accurately assessing these dimensions and their impact on overweight and obesity in LMICs. Furthermore, the hybrid nature of the informal food environment suggests that customer demand is not solely for obesogenic foods, presenting opportunities to incentivise food outlets to offer healthier options. Multiple approaches are needed to address both the retail food environment and customer preferences. Strategies that support informal food retailers in providing healthier options, such as incorporating healthier ingredients, reducing excess fat in fried foods and moderating portion sizes, combined with interventions that stimulate greater demand for healthier food among customers, could offer valuable insights for addressing these challenges and improving public health outcomes.

## Supplementary material

10.1136/bmjgh-2024-017783 online supplemental file 1

## Data Availability

All data relevant to the study are included in the article or uploaded as supplementary information.
