# Supplementary material for: Obesogenicity of food in the informal food retail environment of low- and middle-income countries: a systematic review
Source: BMJ Glob Health. 2025 Dec 19;10(12):e017783. doi: 10.1136/bmjgh-2024-017783 (PMC12716535; doi:10.1136/bmjgh-2024-017783)
Supplement: online supplemental file 1 [file bmjgh-10-12-s001.docx]

**SUPPLEMENT 1: Systematic literature search terms**

**Table 1:** Systematic review search strategy used in EBSCOhost (Global Health).

| **Search number** | **Key search term** | **Search term variation** | **Search yield (N)** |
| --- | --- | --- | --- |
| #1 | Obesity | AB obes* OR overweight OR “body mass index” OR BMI OR unhealthy OR overnutrition | 218225 |
| #2 | Informal | AB informal OR street OR vendor OR market OR “street trade*” OR hawk* OR “mobile food vendor” OR “mobile market” OR kiosk OR “pavement seller” OR “wet market” OR seller OR trad* | 228585 |
| #3 | Food environment | AB food OR “food environment” OR retail* OR “retail environment” OR “obesogenic environment” OR “food system” OR “street food” “built environment” OR "food sector" | 429428 |
| #4 | Low- and middle-income countries and group | AB “Deprived Countries” OR “Deprived Population” OR “Deprived Populations” OR “Developing Countries” OR “Developing Country” OR “Developing Economies” OR “Developing Economy” OR “Developing Nation” OR “Developing Nations” OR “Developing Population” OR “Developing Populations” OR “Developing World” OR “LAMI Countries” OR “LAMI Country” OR “Less Developed Countries” OR “Less Developed Country” OR “Less Developed Economies” OR “Less Developed Nation” OR “Less Developed Nations” OR “Less Developed World” OR “Lesser Developed Countries” OR “Lesser Developed Nations” OR LMIC OR LMICS OR “Low GDP” OR “Low GNP” OR “Low Gross Domestic” OR “Low Gross National” OR “Low Income Countries” OR “Low Income Country” OR “Low Income Economies” OR “Low Income Economy” OR “Low Income Nations” OR “Low Income Population” OR “Low Income Populations” OR “Lower GDP” OR “lower gross domestic” OR “Lower Income Countries” OR “Lower Income Country” OR “Lower Income Nations” OR “Lower Income Population” OR “Lower Income Populations” OR “Middle Income Countries” OR “Middle Income Country” OR “Middle Income Economies” OR “Middle Income Nation” OR “Middle Income Nations” OR “Middle Income Population” OR “Middle Income Populations” OR “Poor Countries” OR “Poor Country” OR “Poor Economies” OR “Poor Economy” OR “Poor Nation” OR “Poor Nations” OR “Poor Population” OR “Poor Populations” OR “poor world” OR “Poorer Countries” OR “Poorer Economies” OR “Poorer Economy” OR “Poorer Nations” OR “Poorer Population” OR “Poorer Populations” OR “Third World” OR “Transitional Countries” OR “Transitional Country” OR “Transitional Economies” OR “Transitional Economy” OR “Under Developed Countries” OR “Under Developed Country” OR “under developed nations” OR “Under Developed World” OR “Under Served Population” OR “Under Served Populations” OR “Underdeveloped Countries” OR “Underdeveloped Country” OR “underdeveloped economies” OR “underdeveloped nations” OR “underdeveloped population” OR “Underdeveloped World” OR “Underserved Countries” OR “Underserved Nations” OR “Underserved Population” OR “Underserved Populations” OR Afghanistan OR Albania OR Algeria OR American Samoa OR Angola OR Armenia OR Azerbaijan OR Bangladesh OR Belarus OR Byelarus OR Belorussia OR Belize OR Benin OR Bhutan OR Bolivia OR Bosnia OR Botswana OR Brail OR Bulgaria OR Burma OR Burkina Faso OR Burundi OR Cabo Verde OR Cape Verde OR Cambodia OR Cameroon OR Central African Republic OR Chad OR China OR Colombia OR Comoros OR Comores OR Comoro OR Congo OR Costa Rica OR Côte d'Ivoire OR Cuba OR Democratic People’s Republic of Korea OR Djibouti OR Dominica OR Dominican Republic OR Ecuador OR Egypt OR El Salvador OR Equatorial Guinea OR Eritrea OR Ethiopia OR Fiji OR Gabon OR Gambia OR Gasa OR Georgia OR Georgia Republic OR Ghana OR Grenada OR Grenadines OR Guatemala OR Guinea OR Guinea Bissau OR Guyana OR Haiti OR Herzegovina OR Hercegovina OR Honduras OR India OR Indonesia OR Iran OR Iraq OR Ivory Coast OR Jamaica OR Jordan OR Kazakhstan OR Kenya OR Kiribati OR Korea OR Kosovo OR Kyrgyz OR Kirghizia OR Kirghiz OR Kyrgyzstan OR Lao PDR OR Laos OR Lebanon OR Lesotho OR Liberia OR Libya OR Macedonia OR Madagascar OR Malawi OR Malay OR Malaya OR Malaysia OR Maldives OR Mali OR Marshall Islands OR Mauritania OR Mauritius OR Mexico OR Micronesia OR Moldova OR Mongolia OR Montenegro OR Morocco OR Mozambique OR Myanmar OR Namibia OR Nepal OR Nicaragua OR Niger OR Nigeria OR Pakistan OR Palau OR Papua New Guinea OR Paraguay OR Peru OR Philippines OR Principe OR Romania OR Rwanda OR Ruanda OR Samoa OR Sao Tome OR Senegal OR Serbia OR Sierra Leone OR Solomon Islands OR Somalia OR South Africa OR South Sudan OR Sri Lanka OR St Lucia OR St Vincent OR Sudan OR Surinam OR Suriname OR Swaziland OR Syria OR Syrian Arab Republic OR Tajikistan OR Tadzhikistan OR Tajikistan OR Tadzhik OR Tanzania OR Thailand OR Timor OR Togo OR Tonga OR Tunisia OR Turkey OR Turkmen OR Turkmenistan OR Tuvalu OR Uganda OR Ukraine OR Uzbek OR Uzbekistan OR Vanuatu OR Venezuela OR Vietnam OR West Bank OR Yemen OR Zambia OR Zimbabwe | 727700 |
| #5 | Combined | S1 AND S2 AND S3 AND S4 | 763 |

**SUPPLEMENT 2**

**Table 1: Summary of included studies.**

| **Author** | **Country** | **Setting** | **Study aims** | **Participants [n)** | **Measure** | **Food environment dimension** | | | **Key findings** | **AXIS appraisal** |
| --- | --- | --- | --- | --- | --- | --- | --- | --- | --- | --- |
|  |  |  |  |  |  | **Availability** | **Accessibility** | **Acceptability** |  |  |
| Akl et al 2023 [49] | Tunisia | School | Documenting the obesogenicity of food environments around schools in Greater Tunis, Tunisia | Food retailers (n=3621) | Food classification: NOVA based classification of retail outlets as 'healthy' 'mixed' or 'unhealthy' | ✓ |  |  | School food environments were dominated by retailers selling unhealthy food. Out of a total of 3621 food retailers surveyed, 27.8% constituted informal outlets. Within this subset, 6% were identified as establishments offering healthy food, while 21.8% were categorised as mixed, providing a combination of healthy and unhealthy food offerings. | High |
| Albuquerque et al 2019 [59] | Tajikistan | Public market | Characterise the street food availability in Dushanbe, Tajikistan | Street food vending sites (n= 800) | Nutrient assessment: Nutritional composition of commonly available street food and beverages.  Food classification: Fruit, beverages, homemade (cooked or 'cooked’, ‘prepared but uncooked’ and ‘uncooked and unprepared) or industrial food. | ✓ | ✓ |  | High availability of energy dense food and beverages in street food vending sites | High |
| Albuquerque et al 2020  [60] | Kyrgyzstan | Public market | Describe the availability and macronutrient composition of street foods in Bishkek, Kyrgyzstan. | Street food vending sites (n=596) | Nutrient assessment: Nutritional composition of commonly available street food and beverages.  Food classification: Fruit, beverages, homemade (cooked or 'cooked’, ‘prepared but uncooked’ and ‘uncooked and unprepared) or industrial food. | ✓ | ✓ |  | The variation in energy, macronutrients, and lipid profile of street food products, both homemade and industrial, reflects heterogeneous culinary practices. | High |
| Albuquerque et al 2020  [62] | Moldova | Public market | Characterise the street food environment in Chi¸sin˘au, Moldova | Street food vending sites (n = 328) | Nutrient assessment: Nutritional composition of commonly available street food and beverages.  Food classification: Fruit, beverages, homemade (cooked or 'cooked’, ‘prepared but uncooked’ and ‘uncooked and unprepared) or industrial food. | ✓ | ✓ |  | Street food offerings include a mix of westernized food options along with substantial servings of energy-dense traditional homemade foods. | High |
| Anggraini et al, 2016 [52] | Indonesia | Neighbourhood | Examine the associations of food store choice with food consumption among urban slum women. | Women (n=188) | Nutritional assessment: Calorie content of consumed food \| Obesity indicator: BMI | ✓ | ✓ |  | Purchasing of food from small shops and street food vendors was associated with the consumption of energy dense- nutrient poor foods | High |
| Basheikh et al 2023 [50] | Tanzania | Neighbourhood (administrative ward) | Assess street vendors' perceptions and attitudes towards healthy meals and the healthiness of their offerings. | Stationary street vendors (n=384) | Mixed methods: Semi-structured questionnaire |  |  | ✓ | Street vendors were cognisant that unhealthy diets are associated with diseases (96%), and that excessive fats and oils in food are detrimental to overall health (99%). However, despite 89% of retailers reporting that they sell healthy meals, only 74% of them emphasised the importance of considering the energy content of the meals they served. | High |
| Berhane et al 2022  [34] | Ethiopia | School | Examine the relationship between the availability of food vendors around schools and adolescents' consumption of unhealthy foods in Addis Ababa, Ethiopia | Schools (n=20),food vendors (n=343),students (n=1200) | Food classification: Categorisation of food as healthy or unhealthy | ✓ | ✓ |  | The number of vendors selling sugar-sweetened beverages, confectioneries, ice cream, and fried foods around the school did not correlate with increased consumption of these items. | High |
| Busse 2023  [35] | Kenya | Neighbourhood | Characterise the food environment of a densely populated informal settlement in Nairobi, Kenya according to the obesogenic properties and spatial distribution of its food vendors. | Food vendors (n=456) | Food classification: NOVA Food classification & Obesity Risk Indicator | ✓ | ✓ |  | Variations existed in the foods sold by different vendors, showcasing a duality of obesogenic risk [high risk, non-protective (34.2%) and low-risk, protective (43.6%)]. | High |
| Carducci et al 2022 [53] | Pakistan | Neighbourhood (village) | To design a survey tool that examines the retail food environment in a peri-urban district of Pakistan and examine key food environment constructs of food outlets by village size. | Formal and informal food outlets (n=1484) | Food classification: Categorisation of food as healthy (nutrient-rich) or unhealthy (nutrient poor) | ✓ | ✓ |  | Food outlets (corner stores and mobile food vendors) sold healthy food alternatives but were also a main source of unhealthy food options. | High |
| Faber et al 2019  [37] | South Africa | School | To assess type, nutrient profile and cost of food items sold by informal vendors to learners. | Informal food vendors (n=92) | Nutrient assessment: Nutrient analysis of corn-based snacks and other food items & Food cost per 100kcal | ✓ | ✓ |  | The majority of available food items were unhealthy choices, and the healthier options tended to be pricier sources of energy. | High |
| Feeley et al 2011  [38] | South Africa | Neighbourhood (village) | To explore the availability of fast food in a rural South African setting. | Fast food vendors (n=18) | Nutrient Classification: Energy and macronutrient analysis of food items | ✓ | ✓ |  | Popular fast food items contained high levels of energy and fat. | High |
| Gewa et al 2021  [36] | Kenya | School | To provide an overview primary school children food environment in two urban settings in Kenya. | Formal and informal food outlets (n=364) and prepared food sources (n=144) | Food classification: Categorisation of food as healthy or unhealthy & Healthy (and Unhealthy) Food Availability Score | ✓ | ✓ |  | More food outlets were located near low-income schools than medium and high-income schools. Additionally, the availability of high-sugar and high-fat foods decreased as school income levels rose. | High |
| Green et al 2020  [39] | Ghana and Kenya | Neighbourhood | To characterise the local foods and beverages sold and advertised in three deprived urban African neighbourhoods. | Formal and informal food outlets (n=1067) | Food classification: Food items were grouped based on a predefined framework of how they would be expected to increase or decrease in availability during the nutrition transition. | ✓ | ✓ |  | On average, food outlets provided a combination of both healthy and unhealthy food options. Informal vendors had a greater variety of healthier foods compared to formal vendors. Among food outlets, small informal sellers were the most prevalent and had the highest proportion of vegetable and fruit stands, despite offering a narrower range of food products. | High |
| Gupta et al 2016  [54] | India | Neighbourhood | To describe the food environment in rural villages and an urban slum setting in India and to analyse the type and quantity of fat in these street foods. | Street food vending site(n=44) | Nutrient assessment: Unbranded snacks assessed for fat composition and oils | ✓ | ✓ |  | Snacks offered by food vendors had high fat content. | High |
| Hernandez Barrera et al 2016  [58] | Mexico | School | Describe the links between the local food environment around elementary schools and schoolchildren’s BMI in two Mexican cities. | Schools (n=60), Students (n=725), mobile food vendors (n=246) food stores (n=103),food establishments (n=177) | Food classification: Unhealthy - high calorie snacks and food, healthy - relatively more nutritious food items, mixed- selling both healthy and unhealthy food items \| Obesity indicator: Body Mass Index (BMI) | ✓ | ✓ |  | The majority of mobile food vendors were categorized as unhealthy. The number of mobile food vendors around schools was positively associated with children's BMI. | High |
| Kagaruki et al 2021  [40] | Tanzania | Public market | To describe the prevalence and correlates of cardio-metabolic risks among regular street food consumers in three districts of Dar es Salaam, Tanzania | Street food consumers (n=560), street food vendors (n=58) | Obesity indicator: Anthropometric measurements & Behavioural risks (alcohol consumption, fruits or vegetable consumption, cooking with oil physical activity and meals eaten outside the home per week |  | ✓ |  | Cardio-metabolic risk factors were prevalent among individuals who regularly consumed street food. This suggests that regular street food consumption is a contributing factor to cardio-metabolic diseases in the general population | High |
| Kagaruki et al 2022  [41] | Tanzania | Public market | To explore the challenges faced by street food vendors, customers and the food and nutrition system, which may prevent the scalability of the healthy plate model in the city. | Street food vendors (n=61), Key stakeholders (n=13) | Qualitative methods: Semi- structured interviews. |  | ✓ | ✓ | Food vendors are willing to sell meals that are inline healthy plate model. However, they are they were concerned that due to food eating preference some of their customers would not agree with the amounts of each food type provided in each plate unless they have been made aware. | N/A |
| Kagaruki et al 2023 [51] | Tanzania | District | Investigate the effectiveness of health information and the healthy plate model interventions on cardio-metabolic risk factors among street food consumers | Street food vendors and their customers (n=366) | Obesity Indicator: BMI | ✓ |  |  | The BMI of individuals in the intervention group, who received health information and subsidized meals based on the healthy plate model, was lower post-intervention compared to baseline. However, this relationship was influenced by age and income. When adjusted for age, BMI was significantly lower in the evaluation phase compared to baseline. Conversely, when adjusted for income, BMI was higher in the evaluation phase compared to baseline. | High |
| Khoe et al 2022  [55] | Indonesia | School | To assess the obesogenic environment in primary schools in Jakarta, Indonesia | School headmasters, physical education teachers, students, school canteen staff, and street vendors around the schools | Qualitative methods: Observation and structured interviews. | ✓ | ✓ | ✓ | The food choices offered in tuckshops and canteens were primarily characterised by their high calorie and sugar content, which was correlated with student food consumption patterns | N/A |
| Khonje et al 2019  [42] | Zambia | Neighbourhood | To examine relationships between consumers’ socioeconomic status, dietary patterns, and use of different modern and traditional retailers. | Households (n=475) | Food classification: Food classified based on level of food processing; whereby ultra-processed foods are considered less healthy than unprocessed food. | ✓ | ✓ |  | The use of traditional retail outlets (includes informal food) was associated with higher vegetable consumption as well as higher consumption of fats and oils, thereby presenting the duality of retail environment. NB* use of all retail outlets was linked to increased consumption of foods with a high sugar content. | High |
| Kroll et al 2019  [43] | South Africa, Ghana | Neighbourhood (Ghana), Transport stop (South Africa) | To understand to what extent food outlets in the local food geography facilitate the consumption of obesogenic foods or of healthier food options. | Households (n= 327 SA; 309 Ghana) \| Formal and informal food outlets in sampling frame (n=83 SA; n=407 Ghana) | Food classification: NOVA Food classification & Obesity Risk Indicator | ✓ | ✓ |  | Increased availability of obesogenic foods in retail stores is associated with a higher intake of these food products within households. In South Africa, over half of the examined food establishments offered obesogenic high-risk foods, whereas in Ghana, the majority primarily offered obesogenic low-risk food items. | Moderate |
| Machado et al 2018  [56] | Brazil | National Survey | To analyse the association between food store type and the consumption of ultra-processed products in Brazil. | 55 970 Household participating in the HBS \| 354 food stores (formal and informal) | Nutrient assessment: Calorie content \| Food classification: NOVA Food classification | ✓ |  |  | The majority of the food items sold by street vendors (83.8%) belong to the NOVA 1 category -unprocessed or minimally processed foods- while only a small portion (9.9%) falls under the category of ultra-processed foods. | High |
| Mensah et al 2022  [44] | Ghana | School (university) | To map and characterise the food-outlets within a Ghanaian university campus and assess the healthiness of the food outlets. | Food retail outlets (n=138) | Food classification: Nutrition Environment Measurement Survey (NEMS) & Categorisation of foods outlets according to non-communicable disease risk NCD Risk ( NCD-healthy (offered healthy food options), NCD-intermediate (contribution o food to BMI and other NCD's is inconclusive), or NCD-unhealthy (ultra-processed foods (UPFs), high-fat, and energy-dense choices). | ✓ | ✓ |  | There was a higher proportion of NCD-unhealthy food outlets offering energy, fat and sugar dense foods, compared to NCD-healthy food-outlets available within the University foodscape. | High |
| O’Halloran et al 2021  [45] | South Africa | School | To compile a comprehensive profile of the home, community and school food environment of primary school aged learners. | Students (n=152) | Qualitative methods: Observation, semi-structured interviews and, photovoice | ✓ | ✓ |  | Learners and their households primarily bought food from nearby 'informal home shop vendors' daily, with 'snacks and fizzy drinks' being the most frequently mentioned items, followed by groceries | High |
| Ogum-Alangea et al 2020  [46] | Ghana | School | To describe types and sources of food in basic schools in urban Accra, and to describe food purchases by pupils. | Students (n=644) | Food Classification: modified Retail Food Environment (mRFEI) score | ✓ | ✓ |  | The majority of food retailers near visited schools (mRFEI=53.4%) offered healthier options, but students still had high exposure to energy-dense foods due to the abundance of unhealthy choices available. | High |
| Pehlke et al 2016  [57] | Guatemala | School | To examine the school food environment at low-income Guatemalan elementary schools and discusses its potential impact on undernutrition and overweight/obesity | School caseta vendors (n=4), school principals (n=4) and children (n=48) | Qualitative methods: In-depth interviews, focus group discussion and direct observation. | ✓ | ✓ | ✓ | Caseta vendors around schools offer mostly unhealthy snacks and sugary drinks with few fruits and vegetables. Concerns about student overconsumption exist, but no interventions have been put in place. | N/A |
| Sekgala et al 2023  [47] | South Africa | Transport stop | To examine minibus taxi driver biochemical parameters, sociodemographic characteristics and lifestyle practices, with a particular focus on street food consumption and the association of these factors with MetS and its components. | Taxi drivers who consume street food (n=185) | Obesity Indicator: Metabolic syndrome (diabetes, high blood pressure, and obesity) diagnosis) |  | ✓ |  | The daily consumption of deep-fried food (meat, fish, snacks) sold by street vendors increased the risk of MetS among taxi drivers. | High |
| Sousa et al 2021  [63] | Bosnia and Herzogovina | Public market | To describe the street food and takeaway food purchases in urban areas of Bosnia and Herzegovina. | Street food (n=194) and takeaway food vending sites, customers (n=755) | Obesity Indicator: Body Mass Index (BMI) | ✓ | ✓ |  | Food purchased from takeaway food vending locations, typically contained higher levels of total fat, trans-fat content, and a higher sodium-to-potassium ratio. In contrast, street food purchases had a greater proportion of saturated fats. | Moderate |
| Sousa et al 2021  [48] | Mozambique | Transport stop | To characterise the extent of processing and nutritional composition of the street foods offered in Maputo, Mozambique. | Street food vending sites (n=810) | Food classification: NOVA food classification | ✓ | ✓ |  | Street food vendors sold both minimally processed (71%)) and highly processed (59%) foods. Mobile street vendors comparatively sold more minimally processed items, while stationary street vendors primarily sold more processed food options. | High |
| Sousa et al 2021  [61] | Tajikistan, Kyrgyzstan, Turkmenistan, Kazakhstan | Public market | To describe street food purchases in cities from Central Asia. | Street food vending sites (n=), customers (n=714) | Nutritional assessment: Nutritional composition commonly available street food and beverages. | ✓ | ✓ |  | A street food purchase contained significant amounts of energy, saturated-fat, trans-fat and sodium, and low levels of potassium. Nearly 30% of customers exceeded 50% of the recommended intake for saturated fat (SFA) from a single street food purchase, while 20.8% exceeded 50% of the recommendation for trans-fat (TFA) | Moderate |
